# Stem Cell-Derived Beta-Cell Therapies: Encapsulation Advances and Immunological Hurdles in Diabetes Treatment

**DOI:** 10.3390/cells15020191

**Published:** 2026-01-20

**Authors:** Sana Waris, Hamna Hameetha Begam, Manyam Praveen Kumar, Zahra Husain I. Abdulrasool, Muthulakshmi Avudaiappan, Alexandra E. Butler, Manjula Nandakumar

**Affiliations:** 1 Research Department, Royal College of Surgeons in Ireland-Bahrain, Adliya 15503, Bahrain; swaris@rcsi-mub.com (S.W.); hbegam@rcsi-mub.com (H.H.B.); pmanyam@rcsi-mub.com (M.P.K.); abutler@rcsi-mub.com (A.E.B.); 2 School of Medicine, Royal College of Surgeons in Ireland-Bahrain, Adliya 15503, Bahrain; 22205762@rcsi-mub.com; 3 Northcote House, University of Exeter, The Queens Drive, Devon, Exeter EX4 4QJ, UK; a.muthulakshmi2484@gmail.com

**Keywords:** pancreatic beta-cells, stem cell-derived beta-cells, encapsulation, immunological

## Abstract

**Highlights:**

- Induced pluripotent stem cell-derived (iPSC) beta-cells hold promise for curing diabetes.
- Challenges for iPSC beta-cells include immune rejection, biocompatibility, and scalability.
- Encapsulation and immune modulation strategies can protect against transplant rejection.
- Personalized therapies with optimized transplant sites reduce biocompatibility/scalability challenges for clinical trials.

**Abstract:**

Diabetes mellitus encompasses a heterogeneous group of metabolic disorders defined by abnormalities in insulin secretion, function, or both. Exogenous insulin therapy has long been the principal treatment strategy for patients with type 1 diabetes and for those in advanced stages of type 2 diabetes. Stem cell therapy has gained significant attention in recent years as a potential curative approach for several life-threatening disorders. In this review, we focus on the use of induced pluripotent stem cells as an alternative source for beta-cell generation, offering a solution to organ scarcity and providing a sustainable supply of insulin-producing cells. We further evaluate current developments in encapsulation technologies and transplantation sites, while noting that the issue of immune-mediated graft rejection continues to be widely debated. The aim of this review is to outline encapsulation techniques and transplantation approaches explored in animal models, and to discuss the risks and challenges anticipated in human clinical trials.

## 1. Introduction

Diabetes mellitus (DM) is one of the most common disease conditions affecting approximately 537 million adults (20–79 years) and children and has resulted in 6.7 million deaths to date. It is estimated that by the year 2045, the total number of people affected by diabetes will be approximately 783 million [1]. DM is classified into two major categories, type 1 DM (T1D), which is associated with immune-driven destruction of pancreatic beta-cells resulting in absolute loss of insulin, and type 2 DM (T2D), characterized by insulin resistance leading to insufficient uptake of insulin by the cells together with partial loss of beta-cell mass through apoptosis [2]. Both T1D and T2D are associated with the risk of atherosclerosis, ischemic heart disease, and nephropathy [3]. These complications are the leading causes of death in diabetic populations.

Prolonged hyperglycemia promotes oxidative stress and overproduction of reactive oxygen species (ROS), leading to pancreatic beta-cell damage and loss of function [4]. Pancreatic beta-cells are particularly susceptible to oxidative stress and damage due to intrinsically low expression of antioxidant genes, which are important factors that lead to apoptosis and a decrease in beta-cell mass [5]. Despite several treatment strategies, cardiovascular disease and other complications are leading causes of morbidity and mortality associated with diabetes, necessitating the need for an alternate approach to cure diabetes [6].

The advent of stem cells has opened up new and promising avenues for regenerative medicine. Stem cells are undifferentiated cells that have self-renewal capacity, allowing them to proliferate into specialized cells under favorable conditions [7]. Stem cells can be distinguished as multipotent or pluripotent. Multipotent stem cells have the capacity to proliferate indefinitely but within a specific lineage. Pluripotent stem cells, conversely, can replicate into every cell type in the human body [8,9]. Pluripotent stem cells are further classified as human embryonic stem cells (hESCs) and induced pluripotent stem cells (iPSCs). Human embryonic stem cells are obtained from blastocysts, the early developmental stage of the embryo. Blastocysts are undifferentiated cells that have paved the way for modern day stem cell therapy [7,10]. Initially, iPSCs were generated through retroviral and lentiviral transduction, which transformed somatic cells into pluripotent cells [11].

The Yamanaka group from Japan was the first group to successfully reprogram somatic cells into iPSCs [9,12]. Due to non-availability of organ donors and the possibility of immune cross-reactions associated with pancreatic or islet transplantation, iPSC-derived beta-cells have garnered the attention of many scientists as an alternate method to treat diabetes. To ensure long-term safety and prevent immune rejection of transplanted iPSCs, advanced cell encapsulation devices can be designed to create immune barriers while allowing the passage of small molecules like nutrients, glucose, oxygen, and insulin. However, several challenges still need to be addressed, including material durability and biocompatibility, sufficient oxygen/nutrient diffusion, prevention of fibrosis formation, scalable and reproducible manufacturing, maintenance of long-term device functionality, and achieving the cellular maturation needed for glucose-responsive insulin secretion. Polyethylene glycol (PEG)-based hydrogels and extracellular matrix (ECM) peptides are the most promising materials being explored for better compatibility and to reduce adverse host responses [13].

Besides therapy, iPSC-derived beta-cells can also be used for disease modeling and drug research [14]. As iPSC-derived beta-cells are produced from patient tissue, the chance of graft rejection is very low; however, their journey from laboratory to clinic still requires assessment of cell viability, functional integration of cellular products, and assessment of safety of cell products in a suitable animal model [15].

**Figure 1:**
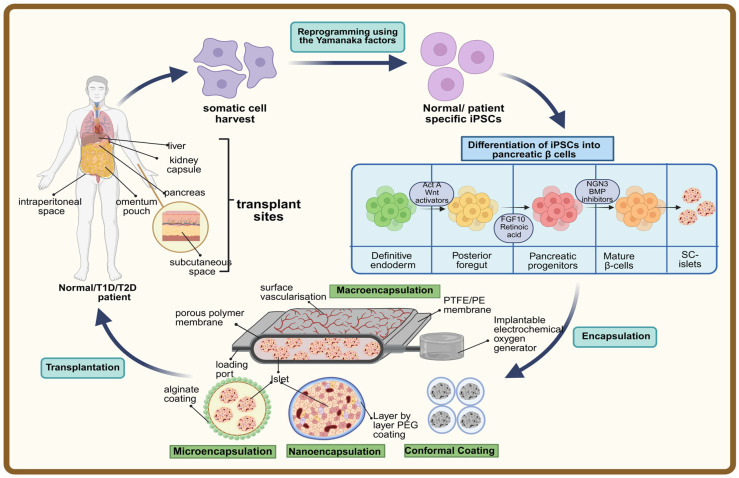
Generation and transplantation of stem cell-derived pancreatic beta-cells for diabetes therapy. Somatic cells from individuals are reprogrammed into induced pluripotent stem cells and subsequently guided through a differentiation process into specified pancreatic progenitors which are further differentiated into mature insulin-producing beta-cells. Stem cell-derived beta-cells can be assembled into pancreatic islet-like clusters, encapsulated, and transplanted into patients.

This review will provide a concise overview of beta-cell generation techniques, materials used, and novel strategies of encapsulation to prevent immune rejection, together with discussion regarding suitable transplantation sites, supported by findings from both preclinical animal models and clinical trials (Figure 1).

## 2. Generation of Pancreatic Beta-Cells from iPSCs

The global surge in diabetes prevalence, coupled with the limitations of insulin therapy and pancreatic/islet transplantation, has propelled cell therapy to the forefront as a promising strategy for potentially curing the disease [16]. Numerous research groups have explored diverse approaches to generate functional insulin-producing beta-cells for diabetes management in both animal models and human subjects; however, these efforts have yet to yield definitive therapeutic success [17].

iPSCs are generated by reprogramming somatic cells into a pluripotent state through the introduction of key transcription factors, such as Oct4, Sox2, Klf4, and c-Myc (the Yamanaka factors) [8,9]. This process erases specialized cell characteristics, enabling their differentiation into any cell type, including insulin-producing pancreatic beta-cells. These iPSCs are valuable for diabetes research and therapy as they allow for patient-specific cell generation, reducing the risk of immune rejection [18]. Advances in reprogramming techniques, such as non-integrating vectors and small molecules, have improved the efficiency and safety of iPSC generation, paving the way for clinical applications.

The successful generation of functional beta-cells is reliant upon understanding the mechanism of cellular differentiation. Multistep protocols have been developed to generate mature, functional beta-cells. These protocols were initially proposed for hESCs, but have also been applied to iPSC lines. The differentiation of iPSCs into pancreatic beta-cells involves several distinct stages, mimicking embryonic development. The first stage is the definitive endoderm-forming stage, wherein iPSCs are directed to form definitive endoderm by exposure to growth factors like activin A and Wingless-related integration site (Wnt) signaling activators. The definitive endoderm is guided to develop into the posterior foregut through factors such as fibroblast growth factor (FGF) and retinoic acid (RA). The next step is formation of pancreatic progenitors, which is a crucial step in beta-cell differentiation. Posterior foregut cells are differentiated into pancreatic progenitors with FGF10 and inhibition of Notch signaling [19,20,21]. Progenitors are driven to commit to the endocrine lineage through exposure to activators like bone morphogenetic protein (BMP) inhibitors, with a focus on expressing transcription factors like neurogenin 3 (NGN3). Finally, the endocrine precursors mature into functional insulin-producing beta-cells under specific cues, including thyroid hormones, glucocorticoids, and extended culture in 3D environments [22].

In 2014, a seven-stage protocol was proposed to differentiate hESCs into beta-cells capable of secreting insulin upon glucose stimulation. The study revealed that pancreatic and duodenal homeobox 1 (PDX-1) and homeobox protein NKX6.1 are the major markers co-expressed in pancreatic progenitor cells and that mature beta-cells showed abundant expression of V-maf musculoaponeurotic fibrosarcoma oncogene homolog A (MAFA) [23]. MAFA is a basic transcription factor present in mature adult beta-cells, responsible for the establishment and maintenance of beta-cell function [24,25].

Another study demonstrated methods for large-scale production of mature insulin-producing human beta-cells from pluripotent cells using sequential modulation of multiple signaling pathways in a three-dimensional cell culture system. The resulting beta-cells expressed key beta-cell markers (33% NKX6-1+/C-peptide+ cells) as well as demonstrating calcium flux in response to glucose [16]. A six-stage protocol was further developed by Veres et al. in 2019 that produced a high number of glucose-sensitive insulin-producing cells, along with enhanced expression of CD49a (also known as ITGA1) as a surface marker for mature beta-cells, and co-expression of insulin, NKX6.1, Insulin gene enhancer protein (ISL-1), paired box 4 (PAX4), and PDX-1 [26]. A recent study reported a six-stage differentiation methodology that produced a high yield of beta-cells with increased insulin secretion and improved glucose sensitivity to meet transplantation requirements. This protocol used a combination of activin A and CHIR99021 for generating PDX-1/NKX6.1-expressing pancreatic progenitor cells. Other growth factors in the cocktail included keratinocyte growth factors (KGF), Smo agonist (SANT1), (2S,5S)-(E,E)-8-(5-(4-(trifluoromethyl)phenyl)-2,4-pentadienoylamino)benzolactam (TPPB), 4-[6-[4-(1-piperazinyl)phenyl]pyrazolo[1,5-a]pyrimidin-3-yl]-quinoline (LDN), and RA. A mixture of Latrunculin A, XXI, Triiodothyronine (T3), Activin receptor-like kinase (ALK5 inhibitor II), SANT1, and RA was used for generation of mature insulin-producing beta-cells [22].

Despite all the efforts made to date, the beta-cells generated do not completely mimic mature insulin-secreting beta-cells. Moreover, the number of cells co-expressing all functional markers was very low. Of note, the iPSCs used for pancreatic differentiation are derived not only from young healthy donors but also from patients with T1D, T2D, and maturity-onset diabetes of the young (MODY) [27,28]. Hence, the stepwise differentiation protocol needs to be optimized for each cell line in order to obtain a sufficient number of insulin-producing cells that can be successfully transplanted into animal models and into humans with minimal immune cross-reactivity.

## 3. Insights into Encapsulation Devices for Transplantation of Beta-Cells

Early studies showed that encapsulation enhances beta-cell function by protecting the cells from immune attack whilst allowing nutrient and insulin exchange, which is particularly crucial in stem cell therapies. The encapsulation approach addresses the challenge of immune rejection in cell-based therapies, where allogeneic cells are often attacked by the immune system. By using encapsulation, transplanted cells can survive without the need for continuous lifelong immunosuppressive drugs, improving both their viability and long-term survival in islet transplantation for T1D [29].

Various encapsulation methods, including macroencapsulation, microencapsulation, nanoencapsulation, and conformal coating, have been explored to enhance stem cell-derived beta-cell transplantation [30]. The success of these techniques largely depends on the materials used, their physicochemical properties, and the site of transplantation, all of which play a critical role in transplant.

### 3.1. Macroencapsulation

Macroencapsulation involves enclosing cell clusters, such as islets, in larger, robust devices made from hydrogels or polymer-based materials. These devices offer structural support and prevent immune cells from directly interacting with the encapsulated cells [31]. Depending on the device size, it can be transplanted under the skin, within the peritoneal space, in the omental pocket, or intravenously [32]. A key advantage of macroencapsulation is its capacity to house larger cell volumes, which is crucial for ensuring sufficient insulin production [30]. A significant development in macroencapsulation is the use of planar implantable devices made from materials like polytetrafluoroethylene (PTFE) and polyester (PE), which support neovascularization. These materials facilitate cell injection through ports and have shown no adverse effects in long-term studies [32]. However, a primary challenge for these devices is the fibrotic response to foreign materials, which can negatively impact cell engraftment, survival, and insulin secretion [30]. To address these challenges, scalable encapsulation devices are being developed that aim to increase cell volume and enhance oxygen and nutrient diffusion. Some designs utilize unique geometries to ensure adequate oxygen supply to cells at the periphery, even in larger grafts. Enhancements like thicker devices help prevent bending and mitigate issues related to cell deposition [33,34]. Despite good mechanical and chemical stability, macroencapsulation devices often struggle with oxygen diffusion due to their larger volume.

Recent advancements, such as flat sheet macroencapsulation devices with porous polymer membranes, are aimed at maximizing oxygen and nutrient diffusion. These innovations have shown improved glucose-responsive insulin secretion with survival rates of up to 10 months, although insulin secretion remained low due to incomplete cell maturation [35]. In a more recent study, a novel encapsulation system was introduced that combines a small, implantable device generating oxygen with a pouch designed for cell containment. This system offers a continuous oxygen supply, supporting high-density encapsulation and minimizing the invasiveness of implantation and retrieval [36]. Additionally, one study [37] demonstrated that incorporating polyvinylpyrrolidone (PVP) into polyvinylidene fluoride (PVDF) membranes enhances glucose diffusion while maintaining immune protection, resulting in improved human islet function in encapsulation devices. The PVDF/PVP devices exhibited higher islet viability and better glucose responsiveness compared to PVDF-only devices while preventing immune cell migration.

A macroencapsulation system developed for pancreatic endoderm cells (PEC-01) incorporates perforated membranes that allow capillary ingrowth, which is vital for improving cell survival by facilitating nutrient and oxygen delivery. However, these membranes do not provide full immune protection and allow immune cell infiltration, necessitating the use of immunosuppression to prevent immune rejection, similar to protocols used in clinical islet allotransplantation [38]. Furthermore, in a study on pancreatic islet encapsulation, alginate hydrogel enriched with perfluorooctylbromide (PFOB) as an oxygen carrier was used within a disk-shaped macrocapsule. After physicochemical testing and biocompatibility evaluation, the macrocapsules were transplanted into diabetic mice and monitored for five weeks, showing promising results in maintaining islet function and survival. Despite the challenges with oxygen transport in macroencapsulation, the use of a high-surface-area-to-volume-ratio spiral geometry significantly enhanced islet viability and function, both in vitro and in vivo, compared to traditional cylindrical designs [39].

### 3.2. Microencapsulation

Microencapsulation involves enclosing cells within small, controlled capsules, allowing precise manipulation of parameters like pore size, thickness, and surface-to-volume ratio to tailor the capsules for specific transplantation sites. Initially introduced in the 1980s for islet transplantation in diabetes, the technique has faced challenges such as immune response, fibrotic encapsulation, and limited nutrient diffusion, which can lead to cell hypoxia and transplant failure. Recent advances in biomaterials, including alginate and polyethylene glycol, aim to improve the biocompatibility, mechanical strength, and functionality of these capsules. Surface modifications and the addition of immunomodulatory molecules are showing promise in reducing immune rejection [40]. Microencapsulation has also been shown to enhance the differentiation of stem cells into islet-like structures, improving the pancreatic islet signature during hiPSC differentiation [41]. In a recent study, adipose-derived stem cells were transduced with miR-375 and anti-miR-7 to differentiate into insulin-producing cells, with their survival rate boosted using a microfluidic system before transplantation. Encapsulating these stem cell-derived beta-cells significantly elevated blood insulin levels in diabetic mice while preserving cell morphology and function [42]. Incorporating immunomodulatory chemokines into encapsulated beta-cells has enhanced glucose-stimulated insulin secretion, reduced inflammation, and prevented fibrotic overgrowth, all without requiring immunosuppressive treatments [43].

Alginate (AG) derivatives remain the most widely used materials for cell microencapsulation due to their excellent biocompatibility and favorable porosity for cell support. Ultra-purified, clinical-grade alginates, free of contaminants like endotoxins and heavy metals, have been shown to prevent inflammatory reactions, ensuring safe islet-cell graft immunoprotection [44]. One study [45] demonstrated the successful encapsulation of stem cell-derived beta-cells in alginate derivatives designed to mitigate foreign body responses whilst providing an immune barrier. These encapsulated implants were successfully transplanted into the intraperitoneal space of diabetic mice, maintaining glycemic control for 174 days without immunosuppression. Incorporating the immunomodulatory chemokine CXCL12 into purified sodium alginate microcapsules enhanced glucose-stimulated insulin secretion (GSIS) and beta-cell function, while also preventing pericapsular fibrosis, enabling long-term glycemic control without immunosuppression in diabetic mice. Strategies to enhance vascularization and incorporate bioactive molecules show promise for improving graft survival, but challenges related to long-term stability and scalability remain obstacles to the broader clinical application of microencapsulation, particularly in T1D.

### 3.3. Nanoencapsulation

Nanoencapsulation is a technique that encapsulates bioactive compounds (BACs) in a protective matrix, which can be liquid, solid, or gaseous, to preserve the substance and control its release. Nanocapsules, typically ranging from 10 nm to 1000 nm in diameter, consist of a core material surrounded by a polymer membrane, offering protection and controlled release, particularly for sensitive substances [46]. Advanced methods like conformal coating or the layer-by-layer (LBL) approach enable the creation of protective nanolayers, making it possible to minimize transplant volume and enhance the transplantation of encapsulated islets into highly vascularized regions, such as the liver, through portal vein infusion [47]. Nanoencapsulation of cells using the LBL technique improves protection and function, offering finer control over release mechanisms compared to macroencapsulation or microencapsulation. Islet transplantation for T1D aims to restore insulin independence and improve glycemic control by infusing donor islets into the liver. However, challenges like islet loss due to hypoxia and the need for lifelong immunosuppression limit long-term success. To address these issues, PLGA-based multifunctional nanoparticles are being developed to reduce the side effects of immunosuppressive drugs and enable real-time monitoring, enhancing transplantation outcomes [48].

### 3.4. Conformal Coating

Conformal coating is a technique applied to enhance the stability and survival of encapsulated cells by applying a thin protective layer over the encapsulating material. This coating improves immune isolation, reduces the risk of fibrosis and immune rejection, and strengthens the material’s mechanical properties, such as flexibility and durability, under physiological conditions. It also regulates the transport of nutrients, oxygen, and waste products, ensuring the continued viability of the cells while shielding them from immune attacks [49]. A promising conformal coating method was developed to overcome the limitations of the intraperitoneal space and minimize hypoxia-induced islet death. By optimizing the hydrogel thickness, diffusion properties were preserved, which is crucial for maintaining islet viability and function. The study found that coated islets maintained viability for up to 100 days with no foreign body response in both in vitro and in vivo syngeneic murine models of islet transplantation. The structural support provided by the coating did not affect islet function compared to uncoated islets [49]. However, single-layer coatings do have drawbacks, including poor long-term stability, susceptibility to membrane turnover, and vulnerability to mechanical and biochemical stress, which can lead to immune attack if the coating is incomplete [50] (Table 1).

In addition to these encapsulation strategies, a research group from California developed an innovative micro- and nanoporous thin-film encapsulation device using the FDA-approved polymer polycaprolactone (PCL) [51]. They demonstrated that encapsulating islets in these PCL devices preserved islet function, provided selective protection from immune cells and cytokines, and showed that islet allotransplantation within these thin-film devices did not trigger a foreign body response, whilst concurrently promoting neovascularization around the device.

**Table 1:** Encapsulation methods for beta-cell transplantation. Summary of microencapsulation, microencapsulation, nanoencapsulation, and conformal coating techniques with their description, advantages, and associated challenges.

Technique	Description	Advantages	Challenges
Microencapsulation	Small gel beads (300–800 µm) that hold one or a few islets. Allows small molecules to pass but blocks the immune cells [52].	High surface area helps with oxygen and nutrient flow [53,54]. Recent advancements incorporate both immune protection and oxygen delivery functions [55]. The coated islets avoided immune attacks without using drugs in mice and kept normal blood sugar levels for a longer time [56].	Requires hundreds to thousands of capsules for humans. Ensuring that all capsules are effective and retrievable remains a challenge. Thicker capsules offer better protection but can reduce oxygen and nutrient flow to the cells. The body might recognize the capsules as foreign, triggering a reaction that forms scar tissue and blocks nutrient access [56,57].
Macroencapsulation	Larger implantable devices (e.g., flat sheets, fibers, chambers) that hold hundreds to thousands of islets or beta-cells [58]. Devices are usually retrievable and may include ports for adding cells or monitoring grafts [59,60].	Can carry more cells than microcapsules, making it better for human-sized treatments. Can be retrieved or refilled surgically. New designs reduce fibrosis and maintain long-term function in animals without immunosuppression [59,60].	If the membrane breaks, many cells can be attacked by the immune system. The cells in the middle might die from low oxygen without blood vessels, especially right after implantation [61]. Very large device volume needed to support human cell doses (e.g., 17 m of hollow fiber for 250,000 islets) [62]. Advanced materials (e.g., zwitterionic coatings) are needed to reduce fibrosis [60].
Nanoencapsulation	Nanoencapsulation involves covering individual islets or cells with an ultrathin, biocompatible membrane, usually just a few nanometers thick [63]. Layer-by-layer technique (LBL) creates a very thin coating that acts like a second skin, allowing nutrients, oxygen, and insulin to move freely in and out of the islet [64].	The thin coating offers effective immune protection without significantly increasing the size [64]. Adding hydrophilic materials like PEG to the coating can reduce immune system reactions [65]. PEG-coated islets transplanted into diabetic rats survived over 100 days with minimal fibrosis [66].	Harmful small molecules, like cytokines and reactive oxygen species (ROS), can still pass through the thin layer [67]. The coating needs to stay stable and protective for a long time in the body [64]. The shape and size of nanoparticles (e.g., rod vs. sphere) can affect how long the coating lasts and where it goes in the body [68].
Conformal coating	Conformal coating involves applying a thin and even layer that tightly wraps around the surface of the islet [63]. The layer is very thin and does not change the size of the islet. It lets oxygen, insulin, and nutrients move in and out [64].	Anti-inflammatory agents can be added to the coating to further protect the islet [67]. The islet remains small and compact, which is useful for transplantation [64].	Even small, uncovered areas can allow immune cells to recognize or attack the islet [64]. Although PEG and similar materials reduce immune response, full immune evasion is hard to achieve [65].

## 4. Biomaterials Used in Beta-Cells or Pancreatic Islet Encapsulation

Biomaterials that enable pancreatic islet or beta-cell encapsulation are a promising approach for the treatment of diabetes (T1D) [69,70].

A biomaterial scaffold platform has been used for beta-cell encapsulation as well as other therapeutic applications including cell carriers, drug delivery, tissue engineering, and flexible electronics, among other applications [71,72,73,74]. These biomaterials can deliver oxygen and growth factors, supporting the survival and function of transplanted beta-cells [75]. Biomaterial-based encapsulation carriers can be categorized by their size into macro-, micro-, and nanodevices [76,77,78] and are divided into natural and synthetic polymers.

### 4.1. Natural Biomaterials

In recent years, hydrogels as biomaterials have been applied to facilitate the integration of transplanted islets within host tissue [79]. These hydrogels are hydrophilic cross-linked macromolecules that form 3D structures; they can be used for beta-cell encapsulation [80] given their precise fine-tunable properties (e.g., porosity and composition), ease of fabrication, and facile biofunctionalization (e.g., tethering of adhesive peptides) [81]. Moreover, they form a natural extracellular matrix (ECM) environment and provide a supportive cell environment that can enhance cell survival and insulin secretion while protecting against an immune response [82]. Natural hydrophilic biomaterials or biopolymers include polysaccharides (alginic acid, agarose, cellulose, chitosan, starch, and hyaluronic acid) and peptides (collagen, poly-L-glutamic acid, and poly-L-lysine), which have numerous applications and can yield derivatives through several different approaches; they also show remarkable potential for micro- and macroencapsulation [83,84].

Alginate is a naturally occurring linear block polysaccharide made up of 1,4′-linked β-d-mannuronic acid (M) and α-l-guluronic acid (G), and is well recognized for its biodegradability, cytocompatibility, biocompatibility, low cost, and easy accessibility [85]. Alginate-based hydrogels have been shown to mimic the structural properties of the natural extracellular matrix (ECM). Since the ECM plays a pivotal role in regulating the cellular microenvironment, incorporating ECM components such as collagen and laminin into biomaterial design has been shown to enhance beta-cell function and support insulin secretion [81]. However, alginate-based hydrogels may contain immunogenic substances, such as endotoxins, polyphenols, or proteins [86], which can lead to fibrosis via fibroblast recruitment. Effective standardized purification procedures are necessary to eliminate these immunogenic components and minimize the host immune response. Another natural polymer is agarose, a linear polysaccharide present in agar. Its application is associated with good biocompatibility and low immune reactivity [87]. Recently, agarose has shown promising outcomes in microencapsulation after islet transplantation in rodents [88]. Furthermore, altering the agarose moiety through concentration changes or combining with other polymers can improve immune defense and structural stability [89].

Chitosan is another potential natural biopolymer composed of β-(1→4)-2-acetamido-d-glucose and β-(1→4)-2-amino-d-glucose unit linkages [90]. Chitosan is widely applied in biomedicine and tissue engineering owing to its inherent antibacterial activity, porous architecture, excellent biocompatibility, biodegradability, and low toxicity [91]. However, its application in islet encapsulation is somewhat restricted due to its low solubility at physiological pH, which may be addressed through innovative chemical modifications [92].

### 4.2. Synthetic Integration with Natural Biomaterials

Numerous researchers have explored the use of natural biopolymers, frequently combined with synthetic polymers, to design novel encapsulation approaches [93]. The βAir macroencapsulation platform from Beta-O2 Technologies incorporates an alginate hydrogel together with a gas-permeable PTFE membrane [94,95].

Besides these strategies, 3D bioprinting technology is in its early stages of emerging as a potential encapsulation method. Marchioli et al. developed 3D-printed hydrogels composed of 4% alginate and 5% gelatin [96]. This method enables layer-by-layer accurate placement of biomaterials to construct engineered, cell-laden scaffolds for islet microencapsulation [97].

Key benefits of 3D bioprinting include the capability to integrate various cell sources and biomaterials, along with accurate control over their positioning and structural assembly [98]. It can also generate high-throughput, clinically relevant multi-component devices within a short period.

### 4.3. Smart Synthetic Biomaterials

In some cases, synthetic materials offer advantages such as high durability, stability, reproducibility, and the ability to fine-tune mechanical and chemical properties [85]. These smart materials utilized in encapsulation are generally organic polymers, such as polyethylene glycol (PEG) [66,99], polydimethylsiloxane (PDMS), polylactic-co-glycolic acid (PLGA), polycaprolactone (PCL), and polytetrafluoroethylene (PTFE). Some of these materials are commonly used as soft hydrogels, but known to degrade during hydrolysis [100], while others require standard manufacturing processes or harsh solvents that are potentially harmful to cells [101].

Despite the significant challenges that persist, collective research efforts with interdisciplinary collaboration across various fields could help overcome these obstacles and facilitate the modulation of biomaterials and longevity of survival of islet tissue through oxygenation into the bio-capsule, enabling the transition of encapsulated islets from basic research to clinical application.

## 5. Immune Modulation Strategies to Prevent Beta-Cell Graft Rejection

A major challenge in cell replacement therapies and transplantation is immunological memory triggered by stress signals within the highly sensitive immune system. The instant blood-mediated inflammatory reaction (IBMIR) initiates allogeneic immune recognition and rejection, activating pre-existing adaptive memory and provoking recurrent autoimmune responses. This process significantly contributes to early graft loss in islet transplantation.

Autoimmune reactions against beta-cells, driven by autoantigens such as GAD65, Tspan7, ZnT8, and IA2, underpin the immune-mediated beta-cell destruction in T1D. Understanding these mechanisms informs immune modulation strategies aimed at inducing tolerance, preserving beta-cell function and preventing T1D progression. Notably, islet autografts also experience an IBMIR similar to xenografts [102], underscoring the universal nature of this response.

Biomaterial scaffolds have been studied to enhance transplantation outcomes by delivering islets or hPSC-derived beta-cells to extrahepatic sites. Both encapsulation methods and non-encapsulating scaffolds, including microporous and fibrous structures, facilitate graft integration, survival, and long-term function [103].

Immune-mediated destruction follows a cascade: inflammation activates self-dendritic cells, which stimulate T cells; these migrate to the graft site, mediating cytotoxicity [104]. Regulatory T cells (TREGs) modulate this response, with natural (nTREGs) and induced (iTREGs) subsets secreting anti-inflammatory cytokines IL-10 and TGF-β, inhibiting dendritic-cell activation and T-cell migration to draining lymph nodes. Antigen-specific TREGs suppress alloimmune responses through these mechanisms [105]. Importantly, iPSC-derived autoantigen-specific TREGs have been shown to suppress autoimmunity and prevent beta-cell destruction by downregulating IFN-γ and ICAM-1 expression in mouse models [106]. 

Genetic modification of mesenchymal stem cells (MSCs) to overexpress ISL-1 transcription factor enhances pancreatic development, promotes angiogenesis, and improves revascularization of transplanted islets, supporting graft survival post-transplantation [107]. Moreover, CRISPR/Cas9-engineered beta-cells secreting IL-10 under glucose control have been developed to modulate immune responses locally [108]. A key strategy involving CRISPR-based hypoimmune engineering entails knocking out the B2M and CIITA genes to suppress HLA class I and II expression, thereby minimizing T-cell recognition, while concurrently overexpressing CD47 to prevent natural killer (NK) cell-mediated cytotoxicity [109]. To mitigate antibody-mediated rejection, CD64 has been overexpressed to bind and sequester IgG Fc domains, thereby shielding cells from antibody-dependent cytotoxicity. When combined with HLA deletion and CD47 overexpression, this approach rendered stem cell-derived beta-cells effectively immune-evasive in both in vitro and in vivo settings [110]. Moreover, genome-wide CRISPR screens have identified additional targets such as CXCL10, whose knockout limits immune cell recruitment, and RNLS, whose deletion enhances resistance to endoplasmic reticulum stress and cytokine-induced apoptosis, collectively improving graft survival and resilience [111,112].

Strategies to improve graft survival include engineering immune-privileged beta-cells derived from stem cells, which demonstrate enhanced survival compared to donor-derived cells [113]. The upregulation of polymorphic HLA class I molecules (HLA-A, -B, -C) on beta-cells facilitates antigen presentation to cytotoxic CD8+ T lymphocytes, leading to targeted beta-cell destruction. Cutting-edge approaches seek to reduce immune rejection by manipulating HLA expression. Donor and recipient HLA matching can be optimized by selectively deleting all HLA alleles except HLA-A2, enabling escape from NK cell-mediated lysis without broad immunosuppression [114]. Consistent with this, human embryonic stem cell lines lacking HLA class I through deletion of β2-microglobulin exons display resistance to CD8+ T-cell-mediated destruction post-transplantation [115].

Overcoming islet autoimmunity remains critical for successful patient-derived beta-cell generation via iPSC technology, as autoantigens expressed by transplanted cells can provoke immune attack even in autologous settings [116,117]. Current immunosuppressive regimens involve induction therapy with agents such as Antithymocyte Globulin (ATG) or IL-2 receptor monoclonal antibodies combined with tacrolimus or sirolimus, though these have significant side effects [118]. Recent strategies have sought to limit toxicity through mild immunosuppressants targeting NK cells.

Brentuximab vedotin (BRE), an anti-CD30 antibody–drug conjugate, has shown efficacy in eliminating undifferentiated iPSCs to prevent teratoma formation without affecting differentiated beta-cells [119]. Furthermore, haplobanks of iPSC lines selected for homozygous HLA haplotypes compatible with broad populations offer a promising strategy for allogeneic transplantation, though manufacturing and immunological challenges remain [120].

## 6. Ideal Sites for iPSC-Derived Beta-Cell Transplant

Identifying the best site for beta-cell transplantation remains challenging as it is important to avoid tissue deposition/agglutination of the capsules implanted at the site. The localization site must maintain oxygen diffusion, hormone exchange, and waste-product removal [30]. Several factors are crucial for prolonged survival and to minimize complications of transplanted islet cells. Rapid revascularization is essential to maintain adequate blood supply for nutrient delivery [121]. Immunoprivileged sites are necessary to alleviate early inflammatory reactions and minimize the rate of islet-cell apoptosis to prevent IBMIR [121,122]. Additionally, the transplantation site should be accessible via a minimally invasive procedure [122,123]. Given the limitations in donor availability and islet-cell quantity, thorough research is required to identify sites that can achieve functional results with the lowest possible number of transplanted islets. 

### 6.1. Liver

The most commonly explored site for beta-cell transplantation is the liver, via the intraportal circulation, for both experimental and clinical applications. The hypothesis underlying the liver site is its role in utilizing insulin and the accessibility of this location [124]. Procedures have evolved from laparotomy cannulation of a mesenteric venous tributary to minimally invasive percutaneous transhepatic administration with fluoroscopic and ultrasonographic aid under local anesthesia [122,123]. This permits repetitive infusions if normoglycemia is not reached during the first transplantation [123]. The liver is often used as a control model in transplantation studies because it requires the least quantity of cells to reverse hyperglycemia. However, the liver location is associated with adverse effects such as short survival time for transplanted beta-cells and unsustained glycemic control, which is why it is not considered an optimal site for transplantation [122,125]. This has been shown in many patients who achieved insulin independence after islet infusion but had to recommence insulin injections in the longer term [123], most patients requiring insulin five years post-transplantation [125]. One study observed 58% insulin independence among 36 recipients across nine centers following portal vein islet infusion, though 76% resumed insulin therapy by two years [126]. Subsequently, another study reported 60% 5-year insulin independence with non-T-cell-depleting immunosuppression [127]; furthermore, one study evaluated 255 patients over 20 years, demonstrating 90% patient survival, 5.9-year median graft survival, and dual anti-inflammatory therapy as the strongest predictor of sustained function [128].

The exposure of the surface antigens of beta-cells in the portal circulation allows for activation of the coagulation and complement pathways, hindering islet survival and function (the IBMIR); this encapsulates the islets in a fibrin clot and further sensitizes innate and adaptive immune reactions by activating Kupffer cells. As opposed to aiming for an immune hyporesponsive site for better survival of beta-cells, the liver shows characteristics of a heightened immune response [122,123,124] with increased plasma levels of inflammatory cytokines such as granulocyte colony-stimulating factor, monocyte chemotactic protein-1, and high-mobility group box 1 (HMGB1) [129]. Strategies to overcome the IBMIR include the administration of different agents to reduce the risk of thrombosis and beta-cell death, such as thrombin inhibitors, heparin, nicotinamide, and low-molecular-weight dextran sulfate [122]. Other disadvantages include portal hypertension, bleeding, thrombosis, hepatic microsteatosis, biliary puncture, hypoxic microenvironment of the portal vein, hypoglycemia unawareness, and the high concentration of toxins and drugs in the liver, including toxic amounts of oral immunosuppressants, which should be managed beforehand [122,123,124,130,131]. The immunosuppressant toxic concentration effect can be eliminated via autologous transplantations as no prior immunosuppressant methods are required [130]. Due to the random distribution of the islet cells throughout the portal system, a histological follow-up for graft assessment is impossible. This can be overcome in the future with further developments in imaging modalities [123].

### 6.2. Subcutaneous Space

Subcutaneous implantation is easily accessible and minimally invasive [132] but has several limitations including the release of insulin into the systemic circulation before reaching the liver and suboptimal vascularization with inadequate nutrient and oxygen supply [117]. Rodent studies testing the effect of pre-vascularization before implantation of islet cells concluded that the rate of reversal of diabetes was superior to the control group. Pre-vascularization is achieved by placing biomaterials under the skin that release growth factors and enhance angiogenesis [117]. For example, the subcutaneous space of the implantation site is pretreated using silicon sheets sewn into vinyl bags; this pouch is inserted between the skin and the muscle layer in mice, thus creating a subcutaneous space, the underlying hypothesis being that it promotes a longer beta-cell lifespan. HiPSC-derived pancreatic endoderm (PE) cells were introduced into the silicon–vicryl pouch in mice, the silicon sheet being removed after 5 weeks; beta-cell mass was better maintained for the 20-week study duration in 60% of recipient mice versus 20% of mice lacking the pouch. This strategy of beta-cell encapsulation is used in ViaCyte, Theracyte, and Nestle encapsulation devices [133]. Similar experiments were performed using device-encapsulated hu-iPSC-PE in nude rats, with contraindicatory results; absence of insulin-positive cells was observed, along with minimal re-establishment of the initial beta-cell mass. The cell mass present primarily consisted of CK19-positive cells forming duct-like structures, surrounded by collagen and connective tissue deposition, indicative of a foreign-body immune response [134]. By contrast, clinical trials involving subcutaneous transplantation of hESC-derived PE cells demonstrated insulin production, though at subtherapeutic levels [129], with another study reporting necrotic islets after allogeneic stem cell transplantation (allo-Tx) administered subcutaneously in humans [123]. These findings highlight the challenges in translating experimental results across different animal models to human subjects.

### 6.3. Intraperitoneal Space

The intraperitoneal space is other potential site for transplantation [135]. Beta-cells derived from human embryonic stem cells were encapsulated into alginate beads implanted into the intraperitoneal space of mice treated with streptozotocin, achieving short-term success up to 174 days without any immunosuppression [43]; the implants induced glycemic correction as demonstrated by C-peptide levels and glucose responsiveness. Mouse pancreatic progenitor cells differentiated into islet-like cell clusters were transplanted into the intraperitoneal cavity of a diabetic rat model, achieving euglycemia for up to 90 days post-transplantation [136]. Intraperitoneal grafting of beta-cells differentiated from iPSCs showed functional competence of beta-cells in non-obese diabetic (NOD) and severe combined immune deficiency (SCID) mice [137]. While intraperitoneal infusion allows for a large number of islet cells to be transplanted, it results in delayed systematic release of insulin and abnormal glucose tolerance [122]. Pericapsular fibrotic overgrowth (PFO) is one of the major factors limiting the long-term success of transplantation, the barrier forming on the capsule surface leading to the depletion of oxygen and nutrients and causing cell death [47,138]; this can be overcome by implanting the microcapsules in the kidney subcapsular space instead.

### 6.4. Omentum Pouch

The omentum is highly vascularized in addition to harboring abundant growth and progenitor factors, such as stromal cell-derived factors and vascular endothelial growth factor; with its easy accessibility, it is a favorable site for islet transplantation [122], mimicking physiological insulin secretion and glucose metabolism as drainage occurs via the portal circulation [123,129]. Though the omentum possesses immunoprivileged characteristics, it still exhibits a strong inflammatory response to foreign bodies; thus, caution is needed during any surgical maneuver for islet transplantation and, if scaffolding or encapsulating is used, to prevent the risk of adhesions and immune rejection [122,123,131]. The team at the Diabetes Research Institute at the University of Miami explored the integration of resorbable scaffolds in combination with islets, thrombin, and autologous plasma in experimental studies, and progressed to clinical trials; laparoscopic transplantation was performed in a patient with T1D with subsequent folding of the omentum to prevent distribution of islets throughout the abdominal cavity, thus allowing for easy removal if indicated. Stable glycemic control was maintained for 6 months post-transplant, with minor deterioration at 12 months post-transplant [131]. Further studies are needed to establish how patient body mass index (BMI), quantity of adipose tissue in the omentum, previous abdominal surgery, and presence of fibrosis and adhesions will affect islet engraftment, function, and viability [131].

### 6.5. Brown Adipose Tissue (BAT)

The well-vascularized and sympathetically innervated brown adipose tissue (BAT) represents an ideal site for islet transplantation as well as islet-cell-loaded macroencapsulation devices, providing adequate oxygenation with minimal shear stress on transplanted cells and ensuring an effective response of beta-like cells. In addition to its function in secreting cytokines and contributing to immune regulation, BAT contains niches of perivascular mesenchymal stem cells [121,139]. The BAT environment contributes to islet survival by delaying immune rejection, decreasing tissue inflammation, and producing proangiogenic factors that improve the overall results of islet transplantation without having a negative effect on BAT function [121,139,140]. However, the effect of adipose tissue heterogeneity on BAT suitability as a transplantation site remains unclear. Further research is needed to compare the immune profile of BAT between healthy individuals and those T1D [140].

### 6.6. Submandibular Gland (SMG)

The histological resemblance of the submandibular gland (SMG) to the pancreas, both containing acinar and ductal tissue, was the driver for testing the SMG as a transplantation site. Diabetic Lewis rats injected with islet cells into the SMG or intraportally were compared; remission from diabetes was seen in only 2/12 rats in the SMG group versus 4/6 in the intraportal group, indicating that the SMG is not a preferential site [125].

### 6.7. Kidney Capsule

The kidney subcapsular site is considered to be the gold standard for experimental stem cell transplantation in rodent models. A relatively low number of cells is needed to reverse streptozotocin-induced diabetes in mice at the subcapsular versus intraportal site [122,131]. Limitations associated with this site include a limited blood supply and the technical difficulty of stretching the subcapsular space for implantation, necessitating a smaller number of implanted cells. Surgical access is invasive with a risk of cross-contamination. During transplantation, exocrine secretions (enzymes) can leak into the graft area, causing local tissue damage, inflammation, or rejection problems. In addition to this, there might be the possibility of diabetic nephropathy in patients, making this less favorable as a transplantation site [122,123].

### 6.8. Pancreas

The pancreas is a suitable environment for transplantation due to its high partial pressure of oxygen, rich vascularization, and physiological response to insulin release. In a mouse model, transplanted beta-cells in the pancreas demonstrated greater metabolic function versus intraportal transplantation. However, the invasiveness of the procedure, the risk of pancreatitis, and the potential for autoimmune attack hypothesized to occur due to previously primed pancreatic lymph nodes in diabetic patients that recognize and target beta-cells are limitations that warrant further research [122,131].

### 6.9. Immunoprivileged Sites

Immunoprivileged sites have the characteristics of an immunosuppressed site where autoimmunity against transplanted cells is absent, thereby offering protection from allorejection or xenorejection without the need for immunosuppressive therapy [122,130,131]. These sites include the anterior chamber of the eye and the brain, thymus, and testes. These sites also have adequate volume for transplanting a clinically functional number of islet cells. Even if an immunoprivileged site lacks adequate volume, it may still be beneficial; transplanting a small number of islets may promote peripheral immune tolerance, with subsequent transplantation into a more acceptable site, thereby enabling maintenance of functionality while benefiting from immunoprivilege [130]. A study showed that co-transplantation of islet cells with Sertoli cells in rats resulted in delayed rejection in the absence of immunosuppression, suggesting benefits from immunoprivileged tissues in the field of stem cell transplantation [131].

Multiple animal studies have investigated allogeneic and xenotransplantation, though few studies have been conducted in humans [129]. Further research is needed to assess the practicality and effectiveness of these immunoprivileged sites.

## 7. In Vivo Cell Therapy in Animal Models

To assess the clinical potential of iPSC-induced beta-cells, it is necessary to first test their functionality in animal models. In the past decade, several groups have used hiPSCs to develop pancreatic beta-cells and evaluated their therapeutic potential in animal models [141,142,143]. The mature pancreatic cells obtained in vitro are defined by their ability to release insulin in response to glucose in a dose-dependent manner in animal models or humans. Several researchers have developed pancreatic endoderm cells expressing beta-cell markers such as NKX6.1 and PDX-1 derived from hESCs and hiPSCs [20,144]. These pancreatic endoderm cells have the capability to further differentiate and mature into insulin-producing beta-cells in vivo. It is suggested that as pancreatic progenitor cells mature into functional beta-cells within the host body, their likelihood of being rejected by the host’s immune system decreases [20]. However, their glucose responsiveness in vivo or in vitro is not the same as adult human islet cells. Moreover, genetic engineering has revealed that newly generated beta-cells still have some resemblance to fetal beta-cells [16,23].

Rezania et al. developed a multistep protocol to generate pancreatic progenitors co-expressing NKX6.1 and PDX-1 which, upon transplantation, matured in vivo into insulin-positive functional beta-cells capable of secreting insulin and reversed hyperglycemia in STZ-induced diabetes in mice. Clinical trials were hindered due to insufficient quantities of cells with adequate insulin secretion and unresolved safety protocols for transplantation [145]. In another study, Haller et al. differentiated hiPSCs into PE cells in vitro which, when transplanted following macroencapsulation into STZ-induced diabetic mice, reversed hyperglycemia and induced normoglycemia. This suggests that PE cells hold great potential for use in a clinical trial [146]. Both pancreatic progenitors and mature insulin-producing beta-cells encapsulated in macro- or microdevices have been explored to assess their functionality in animal models. Beta-cells generated from a seven-stage protocol, populated with insulin-positive endocrine cells expressing key beta-cell markers (MAFA, NKX6.1, NKX2.2), were transplanted into both diabetic and non-diabetic mice. In non-diabetic mice, a significant rise in human C-peptide levels was determined in just two weeks, with higher levels at the four-week timepoint. In streptozotocin (STZ)-diabetic mice, blood glucose levels were lower sixty days post-transplant due to the insulin secreted by the stage 7 transplanted beta-cells [23].

Another study demonstrated successful transplantation of human islet-like organoids that were able to regulate glucose levels in immunocompetent mice for fifty days. These cells were genetically modified to overexpress PD-L1 [147], which creates a local immunosuppressive environment, directly inhibiting infiltrating T cells at the graft site. This reduces reliance on systemic immunosuppression and its associated risks (infection, malignancy, and toxicity) [147]. Millman et al. generated pancreatic beta-cells from T1D patients that expressed all key markers of mature beta-cells. Upon transplantation under the kidney capsule, these cells were able to regulate glucose homeostasis in immunocompromised mice.

The result from these studies could be directed for the development of drugs that would enhance the overall function of beta-cells and reduce stress and death in these cells. As a proof of concept for their potential application in drug screening, a subset of T1D and ND SC-beta-cell lines was treated with three anti-diabetic compounds, each influencing insulin secretion through distinct mechanisms: the sulfonylurea tolbutamide, the GLP-1 receptor agonist liraglutide, and the glucokinase (GCK) activator LY2608204. Treatment with each compound resulted in an average two-fold increase in insulin secretion under both low- and high-glucose conditions compared to the control [148].

Chang et al. used mature hESC-derived beta-cell clusters immunoprotected in a bilaminar synthetic polymer nanoporous device for transplantation in mice. The six-month study revealed viability of transplanted cells along with glucose responsiveness and reversal of diabetic conditions in the animals [149]. Similarly, Vegas et al. demonstrated that alginate-encapsulated mature beta-cells corrected the glycemic conditions in mice without any immune cross-reactivity for 178 days post-transplantation [45]. Sui et al. derived nuclear transfer embryonic stem cells (SCNT) from T1D patients which resembled iPSCs in morphology and function and differentiated them to generate insulin-producing beta-cells that were capable of regulating glucose levels when transplanted in mice lacking endogenous beta-cells. The stem cells obtained from SCNT resemble iPSCs in morphology and function [150]. Several studies using SC-islets, SC-beta-cells, or pancreatic progenitors have confirmed reversal of diabetes in diabetes-induced rodents [151,152,153] or prevention of onset of diabetes [150,154,155,156] (Table 2).

**Table 2:** Summary of differentiation protocols used for study of transplantation of pancreatic beta-cells in animal models.

Year	Author	Type of Cell	Stage	Status of Differentiated Cells	Transplantation in Animal Model	Outcome	Reference
2012	Rezania et al.	ESC	Pancreatic progenitor	ChrA −ve, NKX6.1 +ve, PDX-1 +ve	STZ-induced diabetic mice	Reversed hypoglycaemia in diabetic mice.	[145]
2014	Rezania et al.	ESC	Mature beta-cells	Insulin +ve, MAFA +ve, NKX6.1 +ve, NKX2.2 +ve	Both diabetic and non-diabetic mice	Elevated levels of C-pep in non-diabetic mice. Lowered blood glucose in diabetic mice in 60 days.	[23]
2016	Millman et al.	HiPSCs	Mature beta-cells	C-pep/NKX6.1 +ve, C-pep/PDX-1 +ve	Immunocompetent mice	Regulated glucose levels in immunocompromised mice.	[148]
2016	Vegas et al.	HESC	Mature beta-cells	Insulin +ve C-pep +ve	Immunocompetent diabetic mice (alginate-encapsulated)	Long-term glycemic correction in immunocompetent mice.	[45]
2017	Chang et al.	HESC	Mature beta-cells	C-pep +ve, NKX6.1 +ve, PDX-1 +ve	Diabetic mice (encapsulated in nano- porous material)	Reversal of diabetic condition.	[149]
2018	Sui et al.	HiPSCs	Mature beta-cells (gene-edited cells)	C-pep +ve, MAFA +ve, NKX6.1 +ve	Immunodeficient mice	Induced normoglycemia.	[150]
2019	Haller et al.	HiPSCs	Pancreatic endoderm	C-pep +ve, MAFA/NKX6.1 +ve	STZ-induced diabetic mice (macroencapsulated)	Induced normoglycemia.	[146]
2020	Maxwell et al.	HiPSCs	Mature beta-cells (gene-edited cells)	C-pep +ve, NKX6.1 +ve, PDX-1 +ve	STZ-induced diabetic mice	Reversal of diabetic condition.	[151]
2021	Maxwell et al.	HiPSCs	SC-islets (genetically modified to express PD-L1)	C-pep +ve, NKX6.1 +ve	Immunocompetent mice	Regulated glucose levels for 50 days.	[147]
2024	Hu et al.	iPSCs (hypoimmune	Pseudo-islet	iPSC lines edited/engineered to reduce alloimmune recognition (hypoimmune phenotype)	Immunocompetent diabetic non-human primate	Graft survival and restoration of glycemic control without chronic immunosuppression.	[157]
2023	Shilleh et al.	HPSCs	Insulin-producing beta-like cells (sBCs)	Sox9 +ve, pancreatic progenitor	Kidney capsule or intraperitoneal/omental sites of diabetic mouse	Rapid glucose responsiveness, human C-peptide detection, reversal of hyperglycaemia in many recipients.	[158]

## 8. Clinical Trials in Humans

**Figure 2:**
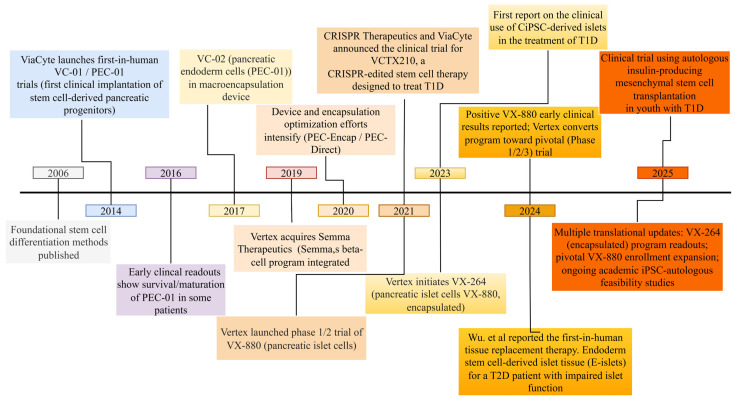
Timeline flowchart summarizing major milestones in the development and clinical testing of stem cell-derived pancreatic beta-cells for the treatment of diabetes [160].

In 2014, following clearance from the US Food and Drug Administration and Health Canada, Viacyte (San Diego, CA, USA) moved forward with the first clinical trial (phase 1/2) of patients with T1D (NCT02239354) using a cellular device VC-01 (Figure 2). The pancreatic progenitors used were produced in large quantities using Good Manufacturing Practices (GMP) and followed all the safety protocols needed for application in stem cell-based therapies. hESC-derived pancreatic progenitors [144] were encapsulated in a microencapsulation device, called Encaptra and designed by Viacyte. T1D patients (aged 18–55 years), both male and female, who had had the disease for at least 3 years were selected and administered with Encaptra (two devices per patient), each containing 10^8^ pancreatic progenitor cells (PEC-01). Study subjects were required to not be on any form of immunosuppression and to exhibit no C-peptide secretion. The Encaptra devices were implanted subcutaneously in the lower back. This study was terminated because of cell necrosis due to hypoxia, the few cells that maintained viability being insufficient [10,159].

In 2017, Viacyte initiated further clinical trials with a modified VC-02 device or PEC-direct (NCT03162926, NCT03163511). These trials were more successful, with the majority of the 15 recipients developing an increase in fasting C-peptide levels with mixed meal-stimulated C-peptide secretion by 6–9 months post-transplantation of PEC-01 cells. A one-year follow-up revealed a decrease in exogenous insulin requirement of 20%.

ViaCyte subsequently modified its PEC-Encap device, in conjunction with the material science company W.L. Gore and Associates (Newark, DE, USA), incorporating polytetrafluoroethylene (PTFE) with both immuno-isolatory and pro-angiogenic properties. With this device, ViaCyte commenced a fourth clinical trial (CI: NCT04678557) in 2020 using up to 12 PEC-Encap devices per recipient, who did not receive any anti-rejection drugs. The trial also included sentinel implants to be removed for histological analysis over a period of 26 weeks. Though the trial has now been terminated, the results are not yet available [161].

In 2018, Viacyte joined with CRISPR Therapeutics (Zug, Switzerland) to develop a cell line via a gene-editing approach that could evade the host immune system [30]. In November 2021, CRISPR Therapeutics and ViaCyte announced their clinical trial for VCTX210, an investigational allogeneic, gene-edited, immune-evasive, stem cell-derived therapy (CyT49 pluripotent human stem cell line that has been engineered to resist immune-mediated destruction—NCT05210530), a CRISPR-edited stem cell therapy designed to treat T1D. An update from February 2023 mentioned that Vertex (Boston, MA, USA)launched VCTX211 (NCT05565248), a collaboration that was initially between ViaCyte and CRISPR Therapeutics, and later involved Vertex Pharmaceutical, alongside VCTX210, with additional gene edits for cell fitness. The VCTX211 combination product consists of two components: PEC211, genetically engineered allogeneic pancreatic endoderm cells using CRISPR/Cas9 technology to enhance immune evasion and cell survival, and a durable, removable perforated device designed for localized delivery and long-term retention of PEC211 cells. Subsequently, Vertex severed ties with CRISPR Therapeutics and the product name changed to CTX211 (marketed by CRISPR Therapeutics). The trial was estimated to be completed by the end of August 2025 [162,163].

Ramzy et al. reported in 2021 [164] implantation of PEC-01 PE cells encapsulated in a microencapsulation device combined with immunosuppressive therapy (ATG) and Mycophenolate Mofeti (MMF) in 15 patients. By week 26, meal-stimulated C-peptide secretion showed a significant increase (*p* = 0.003). Daily insulin requirements decreased by 20% (*p* < 0.001), accompanied by a 13% improvement with time within the target glucose range (time in range, TIR) (*p* < 0.001). Although no participants achieved insulin independence, one individual demonstrated a >50% reduction in insulin use. The implants were well tolerated, with no evidence of teratoma formation [164].

A 24-week study conducted in 2023 by Lian et al. [165] to enhance immune regulation and metabolic control in patients with T2D through intravenous administration of human umbilical cord-derived mesenchymal stem cells (hUC-MSCs) reported enhanced immune response markers, reflected by increased immunoglobulin levels, reduced lymphocyte counts, and an elevated neutrophil-to-lymphocyte ratio. Glycemic indices (HbA1c and fasting blood glucose) and liver/renal function parameters showed no change. Coagulation markers, including D-dimer and fibrinogen, were transiently elevated but returned to normal after treatment [165].

In 2024, Keymeulen et al. [38] transplanted encapsulated PEC-01 PE cells, featuring optimized perforation designs, into recipients maintained on immunosuppression with anti-thymocyte globulin, tacrolimus, and mycophenolate mofetil. By week 26, plasma C-peptide levels rose above 0.07 nmol/L in 4 out of 10 participants. Although insulin requirements decreased, none achieved insulin independence. Glycemic control improved, with TIR (70–180 mg/dL) increasing, and with one participant reaching 85% TIR by month 12 [38].

Vertex launched phase 1/2 clinical trials for T1D in March 2021 (VX-880, NCT04786262). Vertex used the differentiation protocol developed by Melton et al. at Harvard University to generate ESC-derived functional beta-cells along with immunosuppressive therapy. The FDA, however, reduced the proposed dose by 50%, despite the fact that after 90 days, the first patient showed improvement in fasting and meal-stimulated C-peptide levels from 280 to 560 pmol/L, respectively. In addition, HbA1c levels and required insulin dose decreased considerably. The results were released to the global media on 18 October 2021 [166,167]. This trial proved successful as the first T1D patient became insulin-independent and was cured following SC-islet-cell therapy. Furthermore, in 2023, Vertex released data reporting 7 out of 10 patients were able to avoid exogenous insulin completely [168]. In November 2024, Vertex announced that VX-880 from the phase 1/2 trials was progressing to a phase 3 pivotal trial [169], making it the first SC-islet therapy targeting patients with T1D to enter phase 3 clinical trials (NCT04786262). Despite these encouraging outcomes, treatment with VX-880 harbors challenges such as cost and the necessity for long-term immunosuppressive treatment to ensure the survival of the transplanted cells.

To counter the adverse effects of immunosuppressive agents, Vertex launched another therapy for treating T1D, VX-264 (allogeneic human stem cell-derived fully differentiated insulin-producing islets). This SC-islet therapy comes with an encapsulation device, providing an ideal therapeutic approach within a protective and retrievable device. This clinical trial is currently in phases 1/2 (NCT05791201) [170].

In June 2024, Vertex reported preliminary results for VX-880: among the 12 patients enrolled, 11 showed reduction or complete elimination of the need for exogenous insulin. All participants achieved an HbA1c below 7.0% and maintained over 70% TIR on continuous glucose monitoring, despite the reduced/discontinued exogenous insulin. In August 2024, Vertex announced the completion of Part A of the trial, with approval to initiate Part B for which patients are actively being enrolled. However, in early 2025, it was announced that VX-264 did not meet the efficacy endpoint as a clinically relevant increase in C-peptide, indicative of endogenous insulin production, was not achieved. Consequently, VX-264 will not advance to next-phase trials. Meanwhile, Vertex intends to perform further investigations, including analyses of explanted devices, to elucidate the underlying factors contributing to these findings [170].

In 2023, a groundbreaking case study from China reported the successful use of chemically induced pluripotent stem cell-derived islets (CiPSC-islets) (ChiCTR2300072200) to reverse T1D in a 25-year-old female. The procedure involved converting the patient’s own cells into personalized stem cells to grow new islet clusters, which were then transplanted into her abdominal muscles. The patient achieved insulin independence and maintained glycemic control by day 75 post-transplantation. By day 180, the patient’s HbA1c had reduced to 4.6% and was sustained at 4.8% after one year. This marks the first report of clinical use of CiPSC-derived islets for the treatment of T1D [171].

In 2024, Wu, et al. reported the first-in-human tissue replacement therapy using autologous endoderm stem cell-derived islet tissue (E-islets) for a T2D patient with impaired islet function [160]. In this case study, the 59-year-old man with a 25-year history of T2D developed end-stage diabetic nephropathy requiring a kidney transplant in June 2017. Due to significant concerns regarding hypoglycemia and the negative impact of poor glycemic control on the long-term survival of the donor kidney, transplantation with autologous E-islets was performed. The patient underwent a percutaneous transhepatic portal vein transplantation with 1.2 million islet equivalents (IEQs) of E-islets. The first 116-week data revealed significant improvements in glycemic control and provided the first evidence that stem cell-derived islet tissues can rescue islet function in late-stage T2D patients. The grafts were well tolerated with no tumor formation or severe graft-related adverse events [160].

In December 2024, the National Medical Products Administration (NMPA) of China (CXSL2400698) approved the clinical trial application for the developed RGB-5088 islet-cell injection solution [172]. Recently, Sernova Corporation(London, ON, Canada) has successfully tested Cell Pouch technology that involves implantation of a SC-beta-cell-loaded cell pouch into T1D patients, enabling insulin secretion and regulation of blood glucose levels [173].

Another clinical trial started in early 2025 aims to determine the therapeutic efficacy of autologous insulin-producing mesenchymal stem cell transplantation in youth with T1D (NCT06951074). This study aims to generate autologous insulin-producing mesenchymal stem cells derived from adipose tissue for transplant and evaluate the insulin-producing capacity of these cells both in vitro and in vivo.

These clinical trials reflect important milestones in the field of regenerative medicine/stem cell-based therapies. The transplanted islets still, however, face immune rejection under auto-immunogenic or allo-immunogenic settings; thus, addressing immune-related rejection in vivo is imperative.

## 9. Limitations and Future Perspective

Whilst remarkable progress has been made, the major drawback for iPSC-derived beta-cell therapy is the absence of an optimized robust differentiation protocol suitable for every cell line, and developing such protocols will require a huge investment of time and resources [174]. Whilst generation of pancreatic progenitors and insulin-expressing cells has been successfully achieved by many groups, consistently reproducible in vivo maturation of pancreatic progenitors into mature glucose-responsive insulin-secreting beta-cells has yet to be realized. Graft rejection due to immune system activation and tumorigenesis present further hurdles. The immunogenicity of beta-cells derived from autologous iPSCs in the context of T1D was earlier reviewed and it was reported that, despite being patient-derived, these beta-cells can still be recognized by the immune system, suggesting that recurrent autoimmunity may target autologous cells and pose a significant challenge for beta-cell replacement therapies [175]. This was further confirmed in another study where it was demonstrated that autoimmune destruction of beta-cells can recur after transplantation independently of allogeneic rejection and can be detected noninvasively through beta-cell-specific autoantibodies, including GAD65, IA-2, and insulin, as well as autoreactive T-cell responses [176]. These findings indicate that persistent T1D autoimmunity can attack newly transplanted insulin-producing cells, representing a major barrier for both autologous and allogeneic beta-cell replacement approaches. Consistent with this, recent reviews emphasize that immune-mediated injury to beta-cell grafts involves both autoimmune and alloimmune mechanisms, highlighting the need for effective protective strategies, such as encapsulation or localized immunomodulation to enable scalable and durable cell-therapy platforms [117].

Multiple approaches to remove undifferentiated or off-target cells from stem cell-derived beta-cell products, improving both safety and performance, are being studied. Brentuximab vedotin (BRE), an anti-CD30 antibody–drug conjugate, selectively depletes undifferentiated iPSCs to mitigate teratoma risk while preserving differentiated beta-cells [119]. A scalable monoclonal-antibody-based magnetic sorting method was introduced that enriches beta-cell fractions from heterogeneous hPSC cultures without genetic reporters, yielding islet-like clusters with superior beta-cell function and fewer proliferative impurities [177]. Likewise, another study revealed that hiPSC-derived β-like clusters harbor significant proliferative contaminants; bleomycin treatment targeted these cells, boosting in vivo beta-cell performance ~1.7-fold [158]. Self-inactivating vectors, non-integrating adenoviral, or Sendai virus vectors, purified proteins, transposons, modified RNAs, and miRNAs that can inactivate oncogenes immediately after induction of pluripotency may help in reducing the risk of tumorigenesis [178,179]. Aggregation of DNA alterations in iPSCs can increase the risk of tumorigenesis but could be overcome by inducing apoptosis specifically in undifferentiated cells. using, for example, the inducible caspase 9 (iC9) suicide gene [180,181]. CRISPR/CAS9 technology can be used to correct disease-causing mutations [182]. Immunosuppression remains a concern with cell therapy-based treatments [183] but can be addressed using micro- and macroencapsulation devices. Cell hypoxia post-transplantation prior to vascularization [184] can be addressed either by transplanting beta-cells with an inbuilt oxygen supply or using pre-vascularized cells [185,186].

Single-cell transcriptomics combined with spatial multiomic profiling of peripheral blood and graft biopsies can provide deeper insights into immune activation states and reveal cell type-specific signatures that may predict graft acceptance or rejection [187]. High-throughput multiplex analysis of cytokine and soluble biomarker panels may uncover systemic inflammatory profiles associated with early graft outcomes [188]. Ultimately, integrating these multiomic datasets with machine learning tools, such as microRNA-based disease risk scores, can facilitate the development of predictive biomarkers and adaptive trial designs that personalize immunomodulatory regimens according to individual risk profiles. Incorporating these precision-medicine strategies into longitudinal, multicenter studies has the potential to enhance both the efficacy and safety of beta-cell replacement therapies across genetically and immunologically diverse patient populations.

Autologous approaches face hurdles like prolonged manufacturing timelines, high costs per patient, and variable beta-cell maturity from patient-specific iPSCs. Trials often require immunosuppression despite the autologous nature, limiting broad applicability. Ultimately, however, the most critical factor in successful generation of beta-cells from iPSCs or ESCs is the careful handling of cell cultures. Establishment of the ideal composition and quantity of transplanted cells for human patients is needed, together with standardized protocols to generate them [189].

## 10. Conclusions

Although advancements in iPSC-induced beta-cell generation give great hope for reversal of diabetes, more work is needed to optimize protocols and overcome existing hurdles. A suitable encapsulation device and/or immunomodulated cells having the ability to regulate insulin secretion without immunosuppression would pave the way for an effective and reliable therapy to reverse diabetes.

## Data Availability

No new data were created or analyzed in this study.
